# Hypertension self-care practice and its associated factors in Bale Zone, Southeast Ethiopia: A multi-center cross-sectional study

**DOI:** 10.1186/s40545-022-00508-x

**Published:** 2023-02-02

**Authors:** Dagmawi Teshome Tebelu, Tesfaye Assefa Tadesse, Mihiret Shawel Getahun, Yohannes Mekuria Negussie, Abenet Menene Gurara

**Affiliations:** 1 Department of Nursing, Madda Walabu University, Goba, Ethiopia; 2 Department of Nursing, Adama General Hospital and Medical College, Adama, Ethiopia; 3 Department of Nursing, Arsi University, Asella, Ethiopia; 4 Department of Medicine, Adama General Hospital and Medical College, Adama, Ethiopia

**Keywords:** Self-care, Hypertension, Blood pressure control, Bale Zone

## Abstract

**Background:**

Hypertension is a serious threat to public health globally owing to its high prevalence and related complications. It is the main risk factor for cardiovascular disease, kidney disease, eye problems, and death. Self-care practices have been emphasized as a major element in reducing and preventing complications from hypertension. Thus, this study aimed to assess hypertension self-care practices and associated factors in Bale Zone, Southeast Ethiopia.

**Methods:**

A health facility-based cross-sectional study was conducted at three public hospitals from April 1 to May 31, 2021. Data were entered into Epi-Data version 4.6 and exported to Statistical Package for the Social Sciences (SPSS) version 25.0 for analysis. The study participants were characterized using descriptive statistics. The associations between self-care practice and independent variables were modeled using binary logistic regression analysis. Adjusted odds ratios with a 95% confidence interval were used to estimate the association between self-care practice and independent variables. The statistical significance of the association was declared at p < 0.05.

**Results:**

This study involved 405 hypertensive patients, with a response rate of 96.7%. The overall level of good self-care practice was 33.1% (95% CI: 28.6, 37.5). The multivariable logistic regression model showed that age under 65 years (AOR = 3.77, 95% CI: 1.60–8.89), good knowledge of hypertension self-care practice (AOR = 6.36, 95% CI: 2.07–19.56), absence of a depression (AOR = 6.08, 95% CI: 1.24–29.73) and good self-efficacy (AOR = 3.33, 95% CI: 1.12–9.87) were independent predictors of good self-care practice.

**Conclusion:**

The level of good hypertension self-care practice in the study area was low. Hence, it is crucial to expand non-communicable disease control programs and implement public health interventions on self-care for hypertension. Moreover, to enhance hypertension self-care practices, patient-centered interventions are essential.

## Introduction

Hypertension is a significant public health challenge in both developed and developing countries because of its high prevalence and associated complications [1–3]. Systolic blood pressure exceeding 140 mmHg or diastolic blood pressure higher than 90 mmHg as determined by at least two distinct readings taken at different times, or 130/85 mmHg at home, is considered to be hypertension [4]. Nearly a billion people worldwide suffer from hypertension, with two-thirds of those individuals residing in low-income countries [5]. It is also the primary global preventable cause of premature death and disability [3, 6].

One-third of adults worldwide have hypertension, and by 2025, that number is estimated to rise to 1.56 billion adults worldwide, with more than 125 million of those individuals living in sub-Saharan Africa [7–9]. A systematic review and meta-analysis conducted in 2020 estimated that the prevalence of hypertension in Ethiopia is predicted to reach 21.81 percent [10].

Hypertension is the main risk factor for cardiovascular disease, kidney disease, eye problems, and death [5, 11, 12]. Uncontrolled hypertension can result in heart attack, heart failure, and stroke, as well as renal failure, blindness, blood vessel rupture, and cognitive impairment [13]. It contributes to 10.4 million deaths worldwide each year [14]. About 45% of deaths from heart disease and 51% of deaths from stroke are attributable to hypertension [15]. The World Health Organization (WHO) estimates that hypertension complications account for 9.4 million annual deaths, accounting for 17% of all cardiovascular disease-related deaths worldwide[6].

The actual cause of hypertension is uncertain, but research shows that risk can be increased by modifying factors like heavy alcohol use, lack of physical activity, high salt intake, smoking, and a higher body mass index, and avoiding these factors is an essential aspect of self-care practice [6, 9, 16]. Self-care practices are an important and cost-effective non-pharmacologic tool for regulating blood pressure, preventing and minimizing complications, and reducing the ensuing morbidity, disability, and death [17–19]. Taking drugs as prescribed, following a low-salt diet, limiting alcohol use, exercising regularly, managing a healthy weight, avoiding smoking, monitoring blood pressure, lowering stress, and having routine health checkups are all part of self-care practices [4, 20].

Patients must follow self-management protocols for hypertension to meet treatment objectives by raising the quality of life, preventing complications, and spending less on medical care [21–23]. Patients need to start self-care practices before commencing their prescribed medications, as well as continue doing so afterward. Yet, self-care practices are still low in developing countries like Ethiopia [2, 17].

According to previous studies various factors, including sex, age, educational status, place of residence, occupational status, socioeconomic status, time since diagnosis, comorbidity, source of information about self-care and knowledge of disease and treatment, self-efficacy, social support are all associated with components of hypertension self-care practice [24–29].

Although there are few studies on self-care practices among hypertensive patients in Ethiopia, there is no evidence regarding hypertension self-care practices and associated in the study area. Due to this, it was determined that assessing self-care practices and associated factors might help in designing programs and developing strategies for efficient health education and patient empowerment as well as helping close knowledge gaps. Therefore, this study aimed to assess the level of self-care practices and associated factors among hypertensive patients in Bale Zone, Southeastern Ethiopia.

## Specific objectives

- To determine the level of hypertension self-care practices among adult hypertensive patients in public hospitals of Bale zone in 2021.
- To identify factors associated with hypertension self-care practices.

## Methods and materials

### Study design, setting, and period

An institutional-based cross-sectional study was conducted in the Bale Zone, Southeastern Ethiopia from April 1 to May 31, 2021. The Bale Zone is the second largest of the 20 zones in the Oromia Regional State, located 430 km from Addis Ababa, the capital city of Ethiopia. According to the Central Statistical Agency's 2007 population census, the zone's total population is estimated to be 1,402,492, with 688,975 females. The zone has five hospitals, 82 health centers, and 381 health posts. The current study was conducted in three public hospitals, namely Madda Walabu University Goba Referral Hospital (MWU GRH), Bale Robe General Hospital (BRGH), and Dello Menna General Hospital (DMGH).

### Study population and eligibility criteria

The study population consisted of all adult hypertensive patients on follow-up care at the selected public hospitals during the study period and those who met the inclusion criteria. The study included hypertensive patients aged 18 and older who had been on follow-up care for at least 6 months, based on diagnosis codes in the patient hospital records. Patients with cognitive impairment and those who were critically ill during the data collection period were excluded from the study.

### Sample size determination and sampling procedure

The sample size was calculated using a single population proportion formula assuming: *Zα*/2 = 1.96, 95% confidence level, a margin of error (*d* = 0.045), and a proportion of good self-care practice (*p* = 0.299) from a previous study conducted in Harar [30]. By considering a 5% nonresponse rate, the final total sample size becomes 419.

**Fig. 1:**
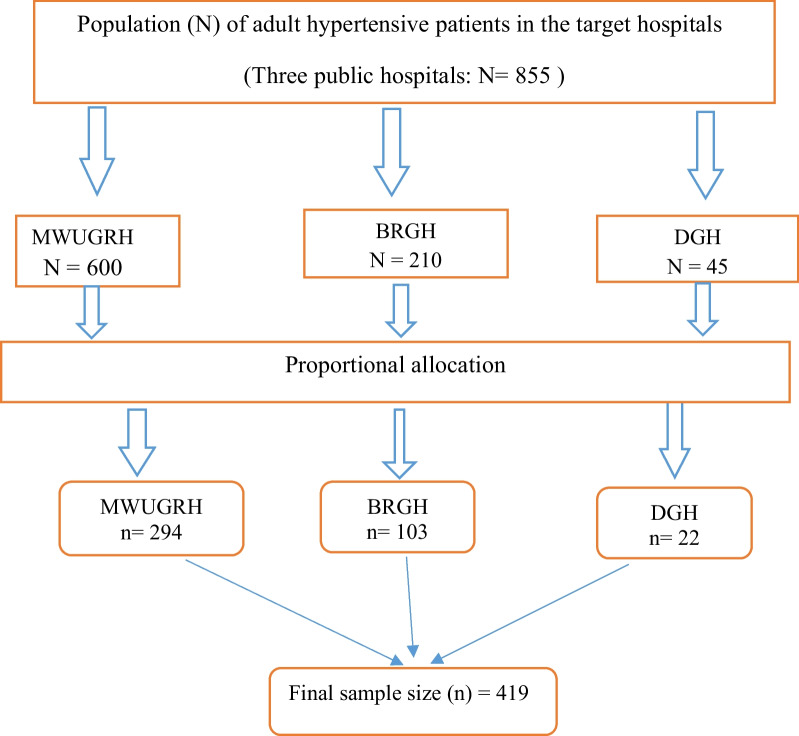
Schematic presentation of sampling technique used to select the study subjects from public hospitals in Bale Zone, Southeast Ethiopia, 2021

The calculated sample size was proportionally allotted to the selected hospitals based on their preceding 3 months’ average client flow for hypertension follow-up during the study period. Accordingly, there were expected to be 855 hypertensive patients across the three hospitals during the study period (i.e., 600 in MWU GRH, 210 in BRGH, and 45 in DMGH) and the calculated sample size was distributed in accordance with this (Fig. 1). To determine the required number of participants from each hospital, we calculated the k-value by dividing the total number of hypertensive patients in the three hospitals by the calculated sample size. Thus, the calculated k-value becomes two. The index participant was then chosen at random from 1 to 2 in each hospital. Accordingly, every second participant was chosen using a systematic random sampling technique until the required sample size was reached.

### Data collection tools and procedures

For data collection, an interviewer-administered structured questionnaire that had been pretested and verified was used. The questionnaires were adapted from validated scales, and published articles [26, 28, 30–37], and were amended for the context of the study. The questionnaire contains socio-demographic characteristics, clinical profiles of the patients, knowledge about hypertension and self-care practice, and psychosocial related factors (depression, self-efficacy, and social support). Six trained nurses, who are Bachelor of Science degree holders, supervised by three Masters of Science degree holder nurses collected the data.

### Study variables

#### Dependent variable

Good self-care practices

#### Independent variables

Socio-demographic variables: age, sex, marital status, occupation, place of residence, educational level, and monthly income. Clinical factors: duration of the disease, comorbidity, family history of hypertension, follow-up miss, and body mass index (BMI). Knowledge of hypertension and self-care. Psychosocial factors: social support, depression, and self-efficacy.

### Study measurements

#### Self-care practice

Self-care practice was measured using the hypertensive self-care activity level effect (H-SCALE) [32]. The scale is divided into six domains (medication adherence, weight management, physical activity, smoking, low-salt diet, and alcohol consumption). When patients scored average or higher on the H-SCALE questions, they were regarded as having good self-care practices.

#### Medication adherence:

Three items containing the number of days in the last 7 days assessed medication adherence. The responses were summed (range: 0–21), and the participants who reported that they had followed these three recommendations on all weekdays were considered adherents (score = 21)[32, 38].

#### Weight management

Weight management was assessed using 10 items based on a 5-point Likert scale ranging from 1 (strongly disagree) to 5(strongly agree) with a sum ranging from 10 to 50. Patients who received 40 out of 50 total points were considered to be good weight management practice adherents [32].

#### Physical activity

Two items were used to assess physical activity. The responses were then added up (range: 0–14). Participants were deemed physically active if they had a score of eight or higher [32].

#### Smoking

Smoking was assessed by one item whether the patients smoked or even a puff in the last 7 days**.** Patients were classified as nonsmokers if they had not smoked or had not taken even one puff in the last 7 days [32].

#### Low-salt diet

Twelve items were used to measure the low-salt diet. After the mean was calculated, a patient is considered adherent to the low-salt diet if they scored ≥ 6(indicating the participant followed low-salt diet practice on 6 out of 7 days) [32].

#### Alcohol consumption

Three items were used to assess alcohol intake. When patients did not drink alcohol at all, they were regarded as abstainers [32, 39].

#### Knowledge

The Hypertension Evaluation of Lifestyle and Management (HELM) scale was used to assess knowledge of hypertension. Patients were deemed to have adequate or good knowledge if their scores were at or above the mean [34].

#### Self-efficacy

Self-efficacy was assessed using the Self-Efficacy to Manage Chronic Disease Scale (SEMCD). The SEMCD is a set of six measures ranging from 1 (not at all confident) to 10 (totally confident). If the patient responds to self-efficacy questions above the mean of six items, he/she was regarded as having good self-efficacy [36].

#### Depression

Depression was screened using the patient health questionnaire-9 (PHQ-9). The PHQ-9 scale goes from 0 (not at all) to 3 (nearly every day) The total number of points ranges from 0 to 27 and if the participant scores ≥ 10, he/she were considered to have depression [37].

#### Social support

Social support was measured by the Oslo Social Support Scale (OSSS-3) containing three items: A four-point Likert scale with a range of 1 to 4 is used to rate the first item. Ratings for the second and third items are given on a five-point Likert scale with a range of 1 to 5. The total range of scores is between 3 and 14. Poor support was defined as 3 to 8, moderate support as 9 to 11, and strong support as 12 or higher [35].

### Data quality management

To assure the quality of the data, data collectors and supervisors were trained for 2 days on research ethics, data collection tools, and procedures. The questionnaire was first prepared in English and then translated into Afan Oromo and back to English to ensure consistency. We pretested the questionnaire on 5% of the total sample (21 hypertensive patients on follow-up) in Ginner Hospital to check for its validity likewise, we adjusted and rearranged some of the questions to ensure clarity, ease of understanding, and cohesion. Investigators and supervisors strictly supervised the data collection process.

### Data processing and analysis

The collected data were coded, cleaned, and reviewed for completeness to reduce data entry errors. The data were then entered into Epi-Data version 4.6 before being exported to the Statistical Package for the Social Sciences (SPSS) version 25.0 for analysis. The normality assumptions were checked to assess the distribution of continuous variables and choose the ideal summary measure. Descriptive statistics were done using frequency, percentage, mean, and standard deviation. Before regression analysis was conducted data were checked for multi-collinearity, normality, linearity, independence of residuals, and outliers, and no significant violations of the regression analysis's assumptions were identified. A bi-variable logistic regression model was used to identify those variables having a *p*-value of < 0.25 to be considered as potential candidate variables for multivariable logistic regression to control confounders. Hosmer and Lemeshow’s goodness-of-fit test was used to check the model adequacy, considering good fit at a *p*-value ≥ 0.05. Afterward, a multivariable logistic regression analysis was performed to identify factors associated with good self-care practices. Adjusted odds ratios (AOR) with a 95% confidence interval were used to report the results. Statistical significance was deemed at a *p*-value of < 0.05.

## Result

### Socio-demographic characteristics of participants

A total of 405 hypertensive patients participated in the study, with a response rate of 96.7%. Of the total participants, 237(51.9%) were males and 212 (52.3%) reside in an urban area. The mean age of the participants was 53 (SD: ± 4.31**)** years. About 309(76.3%) of them were married, and 141 (34.8%) attended college and above (Table 1).

**Table 1:** Socio-demographic characteristics of hypertensive patients in public hospitals of Bale Zone, Southeast Ethiopia, 2021 (n = 405)

Variables	Category	Frequency	Percentage
Sex	Male	237	58.5
	Female	168	41.5
Age (years)	< 65	243	60.0
	≥ 65	162	40.0
Residence area	Urban	193	47.7
	Rural	212	52.3
Marital status	Single	66	16.3
	Married	288	71.1
	Divorced	28	6.9
	Widowed	23	5.7
Occupational status	Student	51	12.6
	Self-employed	148	36.5
	Employed	136	33.6
	Unemployed	24	5.9
	Housewife	46	11.4
Educational status	Unable to read and write	92	22.7
	Able to read and write	81	20.0
	Primary school	53	13.1
	Secondary school	38	9.4
	College and above	141	34.8
Monthly income	< 2500 ETB	64	15.8
	2500–3500 ETB	70	17.3
	> 3500 ETB	271	66.9

*ETB* Ethiopian Birr

### Clinical, therapeutic life change, and self-care practice-related characteristics of participants

In this study, 93 (23%) of the participants had medically confirmed other comorbid diseases, 92 (22.7%) had a family history of hypertension, and 193(47.7%) had a normal body mass index. In terms of the duration of hypertension diagnosis, 239 (59%) of participants were diagnosed less than 5 years ago and 209(51.6%) had a history of missed follow-up. About 254 (62.7%) of the participants had good knowledge about self-care practices for hypertension and 202(49.9%) of them got information about self-care practice from a healthcare professional. Among the participants, 168 (41.5%) had good self-efficacy, 102 (25.2%) had depression, and 148 (36.5%) had strong social support (Table2).

**Table 2:** Clinical, therapeutic life change and self-care practice-related characteristics of participants in public hospitals of Bale Zone, Southeast Ethiopia, 2021 (n = 405)

Variables	Category	Frequency	Percentage
Comorbidity	Yes	93	23.0
	No	312	77.0
Family history of hypertension	Yes	92	22.7
	No	313	77.3
Body mass index (k/m^2^)	Underweight (< 18.5)	49	12.1
	Normal (18.5–24.9)	193	47.7
	Overweight (25–29.9)	101	24.9
	Obese (≥ 30)	62	15.3
Duration of hypertension diagnosis (years)	< 5	239	59.0
	≥ 5	166	41.0
Follow-up miss	Yes	209	51.6
	No	196	48.4
Hypertension self-care knowledge and practice	Poor	254	62.7
	Good	151	37.3
Source of information about self-care practice	Family Members	163	40.2
	Health professionals	202	49.9
	Mass Media	40	9.9
Level of social support	Strong	148	36.5
	Moderate	25	6.2
	Low	232	57.3
Level of self-efficacy	Good	168	41.5
	Poor	237	58.5
Depression	Yes	102	25.2
	No	330	74.8

### Hypertension self-care practices

**Fig. 2:**
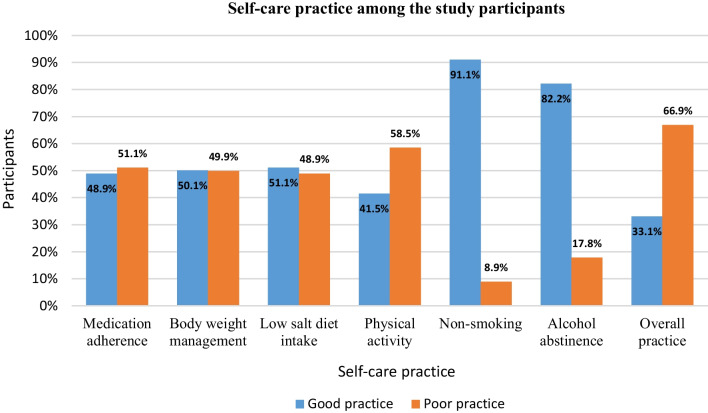
Hypertension self-care practices of the participants in public hospitals of Bale Zone, South East Ethiopia, 2021 (*n* = 405)

The overall level of good self-care practice was 33.1% (95% CI: 28.6, 37.5), determined from the six domains of self-care practice. About 198 (48.9%) of patients had good antihypertensive medication adherence; 203 (50.1%) were good weight management practitioners; 207 (51.1%) practiced a low-salt diet intake, 168 (41.5%) were good practitioners of the recommended level of physical activity; and 369 (91.1%) and 333 (82.2%) were nonsmokers and alcohol abstainers, respectively (Fig. 2).

### Factors associated with hypertension self-care practice

Both bivariate and multivariate analyses were performed to examine the association between independent variables and good self-care practice. Those variables in the bivariate analysis that showed an association with the outcome variable were chosen as candidate variables for multivariable logistic regression analysis at a *p*-value < 0.25.

As a result, in the bivariate analysis, sex, age, place of residence, duration of hypertension diagnosis, knowledge of hypertension self-care practice, depression, self-efficacy, and level of social support were significantly associated with good self-care practice. After adjusting for potential confounding variables using multivariable binary logistic regression analysis, age, knowledge of hypertension self-care practice, depression, and self-efficacy were independent predictors of good self-care practice at a *p*-value < 0.05.

In this study, the odds of having good self-care practice were 3.77 times higher among patients aged younger than 65 years compared with patients who were 65 and olde r(AOR = 3.77, 95% CI: 1.60–8.89). Hypertensive patients with good self-efficacy had 3.33 times greater odds of practicing good self-care practice than those who had poor self-efficacy (AOR = 3.33, 95% CI: 1.12–9.87). The odds of having good self-care among patients with good knowledge of hypertension self-care practice were 6.36 times higher than those with poor knowledge of hypertension self-care practice (AOR = 6.36, 95% CI: 2.07–19.56). Moreover, patients without depression had 6.08 greater odds of having good hypertension self-care practices than patients with depression (AOR = 6.08, 95% CI: 1.24–29.73) (Table 3).

**Table 3:** Factors associated with self-care practices of hypertensive patients in public hospitals of Bale Zone Public hospitals, Southeast Ethiopia, 2021 (*n* = 405)

List of variables	Self-care practice		COR (95% CI)	AOR (95% CI)
	Good (%)	Poor (%)		
Sex				
Male	95 (40.1)	142 (59.9)	1.00	
Female	39 (23.2)	129 (76.8)	2.21 (1.42–3.44)*	
Age (years)				
< 65	105 (78.4)	138 (50.9)	3.49 (2.17–5.61)*	3.77(1.60–8.89)**
≥ 65	29 (21.6)	133 (49.1)	1.00	1.00
Residence area				
Urban	91 (67.9)	102 (37.6)	3.50 (2.26–5.43)*	
Rural	43 (32.1)	169 (62.4)	1.00	
Duration of HTN diagnosis (years)				
< 5	96 (71.6)	143 (52.8)	2.26 (1.44–3.52)*	
≥5	38 (28.4)	128 (47.2)	1.00	
Knowledge of HTN Self-care practice				
Good	100 (74.6)	51 (18.8)	12.68 (7.74–20.79)*	6.36(2.07–19.56)**
Poor	34 (25.4)	220 (81.2)	1.00	1.00
Depression				
No	113 (84.3)	190 (70.1)	2.29 (1.34–3.94)*	6.08(1.24–29.73)**
Yes	21 (15.7)	81 (29.9)	1.00	1.00
Self-efficacy				
Good	105 (78.4)	63 (23.2)	11.95 (7.26–19.67)*	3.33(1.12–9.87)**
Poor	29 (21.6)	208 (76.8)	1.00	1.00
Level of social support				
Low	35 (26.1)	197 (72.7)	1.00	
Moderate	15 (11.2)	10 (3.7)	2.69 (2.12–3.42)*	
Strong	84 (62.7)	64 (23.6)	1.13 (1.48–2.71)*	

*COR* crude odds ratio, *CI* confidence interval, *AOR* adjusted odds ratio, *HTN* hypertension. *Significant at *p*-value < 0.25 in unadjusted logistic regression analysis, **Significant at *p* < 0.05 in adjusted logistic regression analysis, 1.00 = Reference

## Discussion

Self-care is an important non-pharmacological technique that helps to control blood pressure and is beneficial for the treatment and prevention of hypertension. This study assessed self-care practices and associated factors among hypertensive patients in public hospitals in southeastern Ethiopia.

The findings of this study showed that 33.1% (95% CI: 28.6, 37.5) of patients practiced good self-care. Based on the findings of this study around one in three hypertensive patients practiced good self-care practice as advised by the hypertension management protocol. This finding is in line with studies conducted in Harar (29.9%) [30], and West Bengal (37.1%) [40]. This finding was higher than that of studies done in Mekele (20.3%) [41], South Ethiopia (24%) [22], and Debre Berhan (24%) [42]. On the contrary, this finding was lower than studies conducted in Nekemte (68.92%), Gondar (59.4%), Debre Tabor (54.1%) [25], Harar (62.1%) [43] Addis Ababa (51%) [44], Dessie (49%) [26], Saudi Arabia (74.4%) [24] and South India (60.6%) [45]. This inconsistency could be explained by variations in sample size, socioeconomic and cultural differences, levels of understanding of hypertension and its management, differences in the methods and tools used to gather the data, and the methodological disparities between studies. For instance, the WHO STEPwise approach risk factor for non-communicable disease surveillance (STEPS) was used in a Harar study. The Dessie study made use of a tool for practicing hypertension self-care that had 20 items and rated out of four points on a Likert-type scale for each. Moreover, the South Ethiopia study investigated self-care practices using just lifestyle-related characteristics, disregarding medication adherence.

This study showed that the odds of having good self-care practice were 3.77 times higher among younger adults (i.e., patients aged less than 65 years) compared with older adults (i.e., patients who were 65 years and above). This result is supported by studies conducted in India [46], Mekelle [41], Jimma [47], DebreTabor[25], and South, Ethiopia [22]. The possible reason could be the younger the patient, the more likely they are to be educated and driven to exercise good self-care practices and this might also be due to older people's diminishing cognitive function, increased comorbid conditions, and other factors that may make it challenging for them to maintain their lives. This finding, however, contradicts the findings of a study done in Israel [48], Harar [30], Dessie [26], and Addis Ababa [31], which revealed that younger patients were less likely to practice good self-care. This might be due to sample size and participant age categorization variations between the studies.

This study indicated that the odds of practicing good self-care were 3.33 times greater than those with good self-efficacy compared with those who had poor self-efficacy. This result is similar to the findings of a systematic review that reported higher self-efficacy being associated with engagement in self-care behaviors [49]. Studies conducted at the University of North Carolina [33] and Saudi Arabia [50] also showed that Self-efficacy is a strong predictor of good self-care practices. This might be the reason that patients with self-efficacy are aware that their condition is manageable and are confident in dealing with their illness, which enables them to engage in effective self-care practices.

Depression was another significant factor associated with good self-care practices. In this study, patients without depression had sixfold greater odds of having good hypertension self-care practices than patients with depression. This finding is consistent with a study conducted in Deagu, Korea [51]. This might be due to the reason that cognitive side effect of depression that impedes the ability to concentrate and retain appropriate self-care practices.

This study ascertained that patients with good knowledge of hypertension self-care practice were 6.36 times more likely to practice good self-care than those with poor knowledge. This finding is in agreement with studies done in Saudi Arabia [24], Iran [52] Addis Ababa [31], Mekele [41], Debre Tabor [25], and Harar [30]. A study conducted in Debre Brehan also revealed that patients with poor knowledge of hypertension self-care practices were 2.6 times more likely to have poor self-care practices [42]. The justification could be that well-informed patients are aware of their condition, its management, potential complications, and control methods. Patients who are aware of hypertension and its treatment also give self-care practices more heed.

## Limitations of the study

Since the study participants' self-care practices have relied on self-reports, there may be a recall and social desirability bias.

## Conclusion

The study found that the level of good hypertension self-care practice in the study area was low. Age under 65 years, good knowledge of hypertension self-care practice, absence of depression, and good self-efficacy were independent predictors of good self-care practice. Continuous monitoring and follow-up of patient's adherence to the self-management protocol are required, with a greater focus on identifying factors that may hamper patients' adherence to the hypertension self-care protocols. To accomplish this, policymakers should consider these aspects when developing public health initiatives on hypertension self-care and when bolstering ongoing non-communicable disease reduction programs. Moreover, public health facilities should increase their efforts to give patients and families specialized instruction on all components of self-care practice.

## Data Availability

All data and materials are available from the corresponding author without undue reservation.

## Abbreviations

AOR: Adjusted odds ratio
BRGH: Bale Robe General Hospital
COR: Crude odds ratio
CI: Confidence interval
DMGH: Dello Menna General Hospital
ETB: Ethiopian Birr
HTN: Hypertension
HELM: Hypertension Evaluation of Lifestyle and Management
H-SCALE: Hypertension-related Self-Care Activity Level
MSPSS: Multidimensional Perceived Social Support Scale
MWU GRH: Madda Walabu University Goba Referral Hospital
SPSS: Statistical Package for Social Science
